# Enhancing public involvement in trial oversight committees through qualitative research with eight trials facing challenges

**DOI:** 10.1186/1745-6215-16-S2-P78

**Published:** 2015-11-16

**Authors:** A Nicholson, A Daykin, R Macefield, S McCann, G Shorter, M Sydes, C Gamble, A Shaw, JA Lane

**Affiliations:** 1 MRC ConDuCT Hub II for Trials Methodology Research, School of Social and Community Medicine, University of Bristol, Bristol, UK; 2 Formerly Health Services Research Unit, University of Aberdeen, Aberdeen, UK; 3 MRC All-Ireland Hub for Trials Methodology Research, University of Ulster, Coleraine, UK; 4 MRC Clinical Trials Unit, University College London, London, UK; 5 MRC North West Hub for Trials Methodology Research, Institute of Translational Medicine, University of Liverpool, Liverpool, UK

## Background

Trial oversight committees (TOC) including Trial Steering Committees (TSCs) and Trial Management Groups (TMGs) are integral to trial conduct. Patient and public involvement (PPI) in trial design and conduct is frequently stipulated although there is little empirical evidence to optimise roles and inputs. We aimed to use qualitative research to understand the experiences of PPI involvement in TOCs to enhance PPI contributions to trial conduct.

## Methods

The TSC and TMG meetings of eight large trials that were undergoing challenges (e.g. recruitment issues) were observed and audio recorded (n=14) in the QuANTOC^1^ study. Sixty seven members of these TOCs underwent in-depth interviews, which included looking at the role and inputs of PPI members. PPI members interviewed (n=3) also described their experiences and examples of influence on trial conduct.

## Results

The degree to which PPI members were included in TOC influenced their impact on trial conduct. Successful involvement of patients and the public in trial oversight committees can be facilitated through: clarity and transparency of PPI roles; active engagement with the route to and reasons for PPI group membership; and the development and maintenance of support mechanisms during and outside of meetings. Discussions within the trial team are needed to facilitate these components including identifying who is responsible for their completion.

## Conclusion

Greater inclusion of PPI members in TOC can enhance their contributions to trial conduct. The practical suggestions identified can assist in optimising PPI in trial oversight.
